# cerebroViz: an R package for anatomical visualization of spatiotemporal brain data

**DOI:** 10.1093/bioinformatics/btw726

**Published:** 2016-12-15

**Authors:** Ethan Bahl, Tanner Koomar, Jacob J Michaelson

**Affiliations:** Department of Psychiatry, University of Iowa Carver College of Medicine, Iowa City, IA, USA

## Abstract

**Summary:**

Spatiotemporal transcriptomic profiling has provided valuable insight into the patterning of gene expression throughout the human brain from early fetal development to adulthood. When combined with prior knowledge of a disease’s age at onset and region-specificity, these expression profiles have provided the necessary context to both strengthen putative gene–disease associations and infer new associations. While a wealth of spatiotemporal expression data exists, there are currently no tools available to visualize this data within the anatomical context of the brain, thus limiting the intuitive interpretation of many such findings. We present cerebroViz, an R package to map spatiotemporal brain data to vector graphic diagrams of the human brain. Our tool allows rapid generation of publication-quality figures that highlight spatiotemporal trends in the input data, while striking a balance between usability and customization. cerebroViz is generalizable to any data quantifiable at a brain-regional resolution and currently supports visualization of up to thirty regions of the brain found in databases such as BrainSpan, GTEx and Roadmap Epigenomics.

**Availability and Implementation:**

cerebroViz is freely available through GitHub (https://github.com/ethanbahl/cerebroViz). The tutorial is available at http://ethanbahl.github.io/cerebroViz/

**Supplementary information:**

Supplementary data are available at *Bioinformatics* online.

## 1 Introduction

Neuropsychiatric disorders (ND) affect a variety of regions within the brain and exhibit drastically variable ages of onset (Giedd and Rapoport, 2010; Kessler *et al.*, 2005). Understanding the genetic perturbations which underlie distinct phenotypes in ND requires comparing the spatiotemporal gene expression and regulation dynamics of brains developing normally to brains developing abnormally (Kang *et al.*, 2011; Tebbenkamp *et al.*, 2014).

In the last decade, extensive efforts have been made to construct spatiotemporal transcription profiles of the human brain. A substantial portion of these efforts have culminated in the BrainSpan Atlas of the Developing Human Brain (BrainSpan, 2014), which provides publicly available spatiotemporal transcriptomic data from multiple brain regions, spanning fetal development to adulthood. While data collection and analytical techniques in this field have made remarkable strides, critical issues remain in the realm of data visualization. For example, spatiotemporal data is commonly visualized as a heat map. While heat maps are a straightforward approach to convey valuable information, they obscure other potential insights because they do not depict data within an anatomical context. To our knowledge, no tool currently exists to visualize spatiotemporal data for the human brain in an anatomical context.

Here, we present cerebroViz, an R package designed to streamline publication-quality anatomical visualizations of spatiotemporal data in the brain. As input, cerebroViz takes any type of experimental data associated with brain anatomic structures and maps the data to scalable vector graphic (SVG) diagrams of the brain (midsagittal and cortical views), complete with fully customizable coloring and visualization options. While cerebroViz is intended for spatiotemporal gene expression visualization, it is compatible with any data quantifiable at a brain-regional resolution (e.g. expression, methylation, fMRI signal, etc.)

## 2 Implementation

**Fig. 1.**
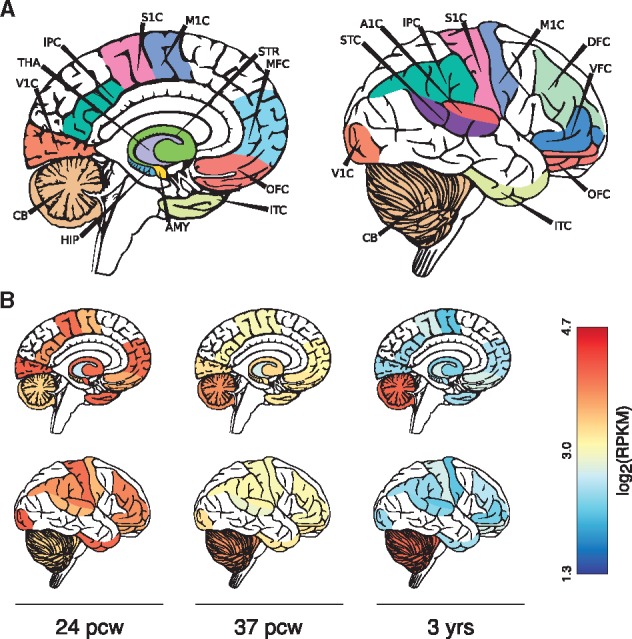
(**A**) Labelled diagrams of cerebroViz output for sagittal (left) and exterior (right) views to illustrate regions used for visualization of *POGZ* expression. Refer to Supplemental Table 1 for full region names; (**B**) Visualization of *POGZ* expression in the brain at three developmental time points (B). pwc = post-conception weeks; RPKM = reads per kilobase per million.

Nearly all of the functionality of cerebroViz is accessible through a single call to the cerebroViz() function. As input, cerebroViz takes a numeric matrix with rows corresponding to brain region and columns corresponding to time point, gene, isoform, replicate, or any other experimental unit. cerebroViz currently supports visualization of thirty brain regions, including those appearing in BrainSpan, GTEx and RoadMap Epigenomics datasets (Supplemental Table S1, Fig. 1A). Behind the scenes, input data is scaled and mapped to a color palette provided by the user. Then, template SVG diagrams of the brain are read into R as XML, altered according to the input data and written out as the final figure in SVG format.

Input data is scaled by one of two methods, depending on color palette type (sequential or divergent). A sequential color palette is ideal for visualizing data as it varies from low to high, while a divergent color palette is recommended for visualizing signed data, such as log2 fold changes. For a sequential color palette, data is scaled linearly. For divergent color palettes, a polylinear scale is employed by juxtaposing two linear scales around the median value. This method ensures median values are mapped to neutral colors, while median-divergent values are assigned to polar colors of the divergent palette. Sequential or divergent visualization may be specified using the divData argument, which is FALSE by default. After scaling input data, cerebroViz() utilizes colorRampPalette() to interpolate color values across the selected color palette.

Additional functionality is built into cerebroViz to enable user customization. For example, the argument customNames allows the user to pass input data with custom region names to avoid renaming the input data to match the standard abbreviation format used by cerebroViz. This is especially important when visualizing multiple datasets where naming conventions have not been harmonized. Other features allow users to hide the figure legend, add a cross-hatch pattern to regions with missing data, and to clamp outlier data in the scaling step. The full functionality of cerebroViz, including a tutorial on creating animated GIFs (e.g. to depict temporally dynamic gene expression), is described in the package vignette.

## 3 Application

A recent study explored the role of the gene *POGZ* in intellectual disability and autism (Stessman *et al.*, 2016), and this analysis included an examination of spatiotemporal expression patterns of *POGZ* in BrainSpan and GTEx (GTEx Consortium, 2013). The data were visualized with boxplots, which, along with heatmaps, are the standard method for visualizing this type of data. To demonstrate a practical application of cerebroViz, and to emphasize the value of anatomical context in these visualizations, we have created figures of *POGZ* expression for ages 24 post-conception weeks (pcw), 37pcw (birth) and 3 years (Fig. 1B).

Figure 1 shows *POGZ* expression for both a sagittal and exterior view of the brain across three time points. The first time point (24pcw) shows a global trend of elevated *POGZ* expression. The second time point (37pcw) shows a decrease in global expression to median levels, with an increase specifically in cerebellar expression of *POGZ*. The third time point (3 years) shows a reduction in global expression of *POGZ*, except in the cerebellum where expression is elevated. The cerebellum plays a role in motor coordination, and this study found that *POGZ* mutation carriers often had gross motor phenotypes; consequently the pronounced expression in the cerebellum is of interest. As demonstrated in this example, anatomical context can be a useful tool to drive home the region and time-specific effects—along with potential functional interpretations—of gene expression in the brain.

## 4 Conclusion

cerebroViz is a user-friendly R package for visualizing spatiotemporal and other region-resolved data of the human brain within an anatomical context. The package, along with a detailed tutorial demonstrating its features, is available at https://github.com/ethanbahl/cerebroViz.

## Funding

This work has been supported by a National Institutes of Health award (R01-MH105527).

*Conflict of Interest*: none declared.

## Supplementary Material

Supplementary Data
