# Supplementary material for: cerebroViz: an R package for anatomical visualization of spatiotemporal brain data
Source: Bioinformatics. 2016 Dec 15;33(5):762–3. doi: 10.1093/bioinformatics/btw726 (PMC5870797; doi:10.1093/bioinformatics/btw726)
Supplement: Supplementary Data [file btw726_supp.docx]

| Abbreviation | Brain region |
| --- | --- |
| A1C | primary auditory cortex |
| AMY | amygdala |
| ANG | angular gyrus |
| BS | brainstem |
| CAU | caudate |
| CB | cerebellum |
| CNG | cingulate gyrus |
| DFC | dorsolateral prefrontal cortex |
| FL | frontal lobe |
| HIP | hippocampus |
| HTH | hypothalamus |
| IPC | inferior parietal cortex |
| ITC | inferior temporal cortex |
| M1C | primary motor cortex |
| MED | medulla oblongata |
| MFC | medial prefrontal cortex |
| OL | occipital lobe |
| OFC | orbital prefrontal cortex |
| PL | parietal lobe |
| PIT | pituitary gland |
| PUT | putamen |
| PON | pons |
| S1C | primary somatosensory cortex |
| SN | substantia nigra |
| STC | superior temporal cortex |
| STR | striatum |
| TL | temporal lobe |
| THA | thalamus |
| V1C | primary visual cortex |
| VFC | ventrolateral prefrontal cortex |

**Table 1**. Thirty brain regions supported by cerebroViz. The left column shows default naming conventions used by cerebroViz for regions listed in the right column.
